# 原发性骨髓纤维化转化Ph^+^急性淋巴细胞白血病1例

**DOI:** 10.3760/cma.j.issn.0253-2727.2023.07.006

**Published:** 2023-07

**Authors:** 燕平 刘, 芳 李, 晗 朱, 建勇 李, 扣荣 缪

**Affiliations:** 江苏省人民医院（南京医科大学第一附属医院）血液科，南京 210029 The First Affiliated Hospital of Nanjing Medical University, Jiangsu Province Hospital, Nanjing, 210029, China

患者，女，33岁。2020年7月因“阑尾炎”手术前查血常规WBC 31.79×10^9^/L，中性粒细胞27.23×10^9^/L，嗜酸性粒细胞0.45×10^9^/L，HGB 182 g/L，PLT 470×10^9^/L；外周血骨髓增殖性肿瘤相关基因检测JAK2 V617F突变阳性，CARL、MPL及JAK2 Exon 12突变均阴性。骨髓象：有核细胞增生明显活跃，粒系占80.5％，粒红比例明显增高，成熟分叶核易见，可见分叶过多、胞质颗粒增多；外周血涂片可见中晚幼红及中晚幼粒细胞。骨髓活检病理：增生极度活跃（>90％），粒红比例明显增大；粒系增生明显活跃，各阶段细胞可见，核左移，中幼及以下阶段为主，嗜酸性粒细胞易见；纤维组织增生，网状纤维染色MF2级。JAK2 V617F突变比例95.6％；未检出BCR-ABL1融合基因转录本。B超示脾长径18 cm、肋间厚6 cm。LDH 522 U/L（参考值125～250 U/L）。临床诊断为原发性骨髓纤维化明显纤维化期（IPSS 2分，中危-2）。予口服羟基脲及芦可替尼治疗。

2022年4月患者开始出现全身散在瘀斑伴全身乏力，至我院门诊查血常规：WBC 33.64×10^9^/L，HGB 62 g/L，PLT 11×10^9^/L。骨髓象：原始细胞占72.4％，POX染色阴性，PAS染色阴性。流式细胞术免疫分型示异常细胞占77.2％，表达cCD79a、CD34、CD33、CD13、CD19、CD10、CD22、CD20、CD81、CD66c、CD38及CD56，提示伴CD13^+^CD33^+^急性B淋巴细胞白血病；骨髓活检示MF 3级，BCR-ABL融合基因FISH检测阳性（融合探针信号267/300），56种白血病融合基因检测BCR-ABL P190阳性，AML/MPN二代基因测序JAK2 V617F阳性（20.02％）；染色体核型为44, XX, add（3）（q12）, add（4）（q35）, −7, −9, t（9;22）（q34;q11）, add（13）（q14）[4]/46, XX[16]。患者病情进展为Ph染色体阳性急性B淋巴细胞白血病。2022年5月18日起予地塞米松10 mg/d预处理治疗，1周后开始服用奥雷巴替尼40 mg隔日1次并联合长春新碱每周1次。2022年6月6日复查骨髓达形态学缓解，BCR-ABL P190 0.34％。后行腰椎穿刺并鞘内注射，继续口服地塞米松联合奥雷巴替尼治疗。

2022年7月患者因“脾梗死”于外院行“脾切除术”。2022年8月复查血常规：WBC 45.6×10^9^/L，中性粒细胞78％，HGB 159 g/L，PLT 1730×10^9^/L，骨髓涂片未见原幼淋巴细胞；BCR-ABL P190 0.0357％，JAK2 V617F 53.26％。予羟基脲及芦可替尼控制白细胞及血小板。

患者于2022年8月行异基因造血干细胞移植（供者为胞姐，HLA 10/10相合，O+供A+），移植过程顺利。随访至今病情稳定。

